# Genetic predisposition to smoking is associated with risk of rheumatoid arthritis: a Mendelian randomization study

**DOI:** 10.1186/s13075-020-2134-1

**Published:** 2020-03-06

**Authors:** Yu Qian, Lingzhi Zhang, David J. H. Wu, Zhijun Xie, Chengping Wen, Yingying Mao

**Affiliations:** 1 School of Public Health, Zhejiang Chinese Medical University, Hangzhou, 310053 Zhejiang China; 2 University of Minnesota Medical School, Minneapolis, MN 55455 USA; 3 Institute of Basic Research in Clinical Medicine, Zhejiang Chinese Medical University School of Basic Medical Sciences, Hangzhou, 310053 Zhejiang China; 4 Department of Epidemiology & Biostatistics, Zhejiang Chinese Medical University School of Basic Medical Sciences, Hangzhou, 310053 Zhejiang China

**Keywords:** Mendelian randomization, Rheumatoid arthritis, Single nucleotide polymorphism, Smoking

## Abstract

**Background:**

Although observational epidemiological studies have found that smoking is positively associated with risk of rheumatoid arthritis (RA), assessing the causality of this relationship has remained elusive because conventional observational studies are susceptible to bias such as confounding and reverse causation. Here, we applied the Mendelian randomization (MR) approach to examine the potential causal relationship between smoking and risk of RA.

**Methods:**

Summary statistics data for RA were obtained from a meta-analysis of genome-wide association studies (GWAS), including 14,361 RA cases and 43,923 controls of European ancestry. The instrumental variables (IV) and the genetic association estimates for smoking initiation and lifetime smoking were obtained from a GWAS meta-analysis including 1,232,091 individuals and a GWAS of 462,690 individuals of European ancestry, respectively. MR analyses were performed using the inverse-variance weighted (IVW) method and supplemented with the weighted-median method. Potential pleiotropy was assessed using the MR-Pleiotropy RESidual Sum and Outlier (MR-PRESSO) test and MR-Egger regression. Sensitivity analyses were further performed to test the robustness of the association.

**Results:**

We found that compared with never smokers, genetic predisposition to smoking initiation was positively associated with risk of RA (odds ratio (OR) = 1.32, 95% confidence interval (CI) = 1.15–1.52, *P* = 9.17 × 10^−5^ using the IVW method). Similarly, genetically predicted lifetime smoking was associated with an increased risk of RA (OR = 1.55, 95% CI = 1.13–2.14, *P* = 0.007). Sensitivity analyses using alternative MR methods and different sets of IVs produced similar results, suggesting the robustness of our findings.

**Conclusions:**

These results provide support for a causal association between smoking and increased risk of RA. Further studies are warranted to explain the underlying mechanisms of smoking in the development of RA.

## Background

Rheumatoid arthritis (RA) is a chronic autoimmune disease that causes cartilage and bone damage, functional loss, and associated comorbidity. It affects about 1% of the population and is more prevalent in women than in men [1]. Although the etiology of RA remains unclear, it is thought that the interplay of genetics, environment, and the immune system plays a major role in its development [2].

Historically, observational epidemiological studies have investigated smoking as an important modifiable risk factor for RA. Though there are inconsistent findings, evidence from these observational studies generally supports a positive association of smoking with risk of RA. For example, a meta-analysis of five cohorts and 11 case-control studies involving 584,455 individuals showed a 40% higher risk of RA among ever smokers compared to never smokers [3]. Another dose-response meta-analysis of three cohorts and seven case-control studies found that compared to never smokers, the risk of RA increased by 26% for those who smoked 1 to 10 pack-years and 94% for those with more than 20 pack-years [4]. However, conventional observational studies generally rely on self-reported information and are susceptible to potential confounding and reverse causation. Therefore, the causal nature of this association remains elusive.

Mendelian randomization (MR) offers a way to investigate the nature of the relationship between smoking and risk of RA. It utilizes instrumental variables (IV) such as genetic variants that act as proxies for environmental or behavioral factors to determine whether an observational association between a risk factor and an outcome is consistent with a causal effect [5]. Because genetic variants are naturally and randomly assorted during meiosis, confounding factors are anticipated to be equally distributed among different genotypes. Therefore, results from MR studies are less prone to confounding and reverse causation bias. In the present study, we applied a two-sample MR approach to examine whether genetic predisposition to smoking was associated with risk of RA.

## Methods

### Data sources

**Fig. 1:**
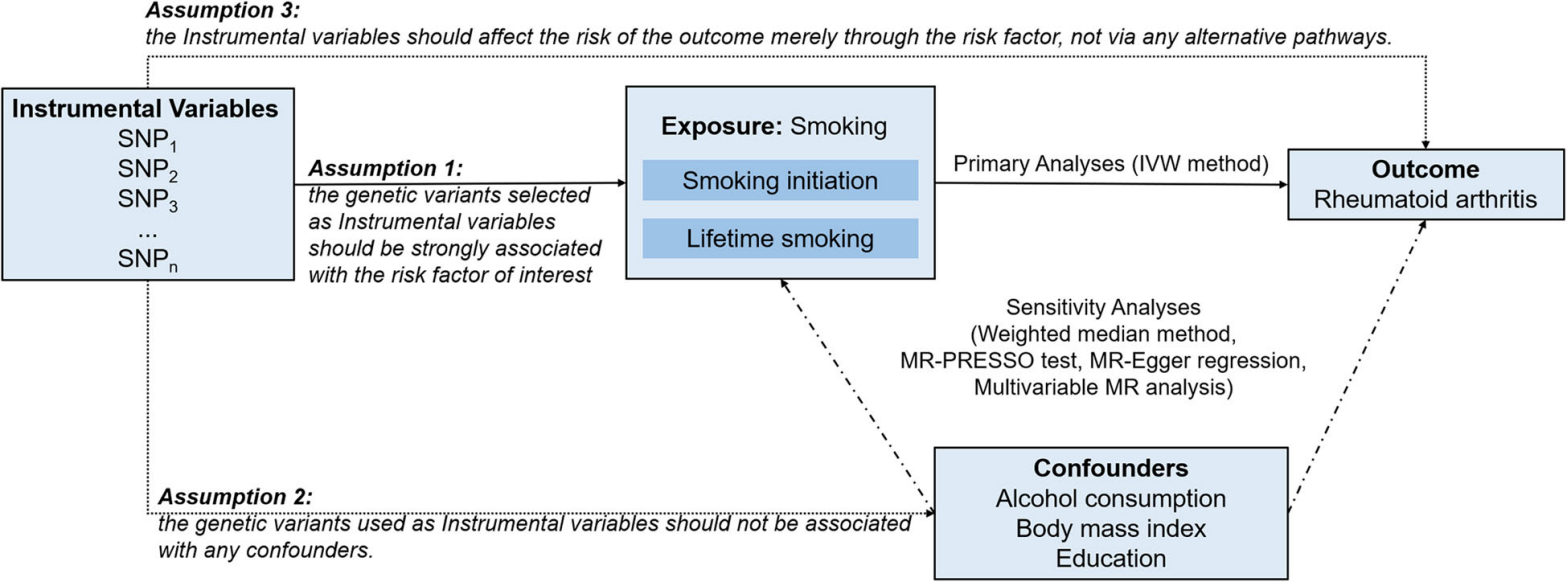
An overview of the study design. IVW, inverse-variance weighted; MR, Mendelian randomization; MR-PRESSO: MR-Pleiotropy RESidual Sum and Outlier; SNP, single nucleotide polymorphism

We performed the MR analysis with summary statistics data from published genome-wide association studies (GWAS). An overview of the study design is shown in Fig. 1. Summarized data (effect size estimates and their standard errors) for the associations between genetic variants and risk of RA was obtained from a meta-analysis of genome-wide association studies (GWAS), including 58,284 individuals of European ancestry (14,361 RA cases and 43,923 controls) [6]. Detailed information of the study has been described elsewhere [6]. Briefly, all cases were diagnosed by a board-certified rheumatologist or met the 1987 criteria of the American College of Rheumatology for diagnosis of RA [7].

### Selection of instrumental variables

The genetic IVs related to smoking initiation (ever being a regular smoker vs. never being a regular smoker) were obtained from a GWAS meta-analysis, including 1,232,091 individuals of European ancestry [8]. This study identified 378 conditionally independent single nucleotide polymorphisms (SNPs) associated with smoking initiation at genome-wide significance threshold (*P* < 5 × 10^− 8^). All 378 SNPs together accounted for 2.3% of the variance in smoking initiation. However, 34 SNPs were not available in the summary statistics data for RA. A proxy variant in linkage disequilibrium (*r*^2^ > 0.9) with the specified genetic variant was identified for 23 of the missing SNPs. Therefore, a total of 367 SNPs were used as IVs for smoking initiation in the present study.

As an additional analysis, we used 126 independent SNPs associated with lifetime smoking at genome-wide significance from a GWAS of 462,690 individuals of European ancestry [9]. The lifetime smoking index captured individual aspects of smoking status, smoking duration, heaviness, and cessation among ever smokers. These SNPs were broadly distinct from the SNPs associated with smoking initiation, and strongly related to the control of lung cancer [9]. Among them, 124 (including 5 proxy variants) were available in the summary statistics for RA.

### Statistical analyses

Statistical analyses were performed using the MendelianRandomization [10], and MR-PRESSO [11] packages in R software version 3.6.0 (https://www.r-project.org/), unless otherwise noted. All estimates were reported with two-tailed *P* values. In the main analysis, we used the inverse-variance weighted (IVW) method based on a random-effects model and supplemented with the weighted-median method [12]. To test for potential pleiotropy, we performed the MR-Pleiotropy RESidual Sum and Outlier (MR-PRESSO) test [11] and MR-Egger regression [13]. Each of the SNPs used as IVs we scanned for its potential secondary phenotypes using the GWAS catalog (http://www.ebi.ac.uk/gwas, accessed on November 20, 2019), and sensitivity analyses were further performed excluding the SNPs associated with traits other than smoking. *F*-statistics were calculated to evaluate the strength of the IVs [14].

In addition, since smoking initiation is genetically correlated with education attainment (*r*_g_ = − 0.40), alcohol consumption (*r*_g_ = 0.34), and body mass index (BMI) (*r*_g_ = 0.12) [8], we performed multivariable MR analysis including the SNPs associated with these exposures, along with SNPs for smoking initiation, to test for whether the association between smoking and RA remained statistically significant after adjustment for confounders [15].

## Results

**Fig. 2:**
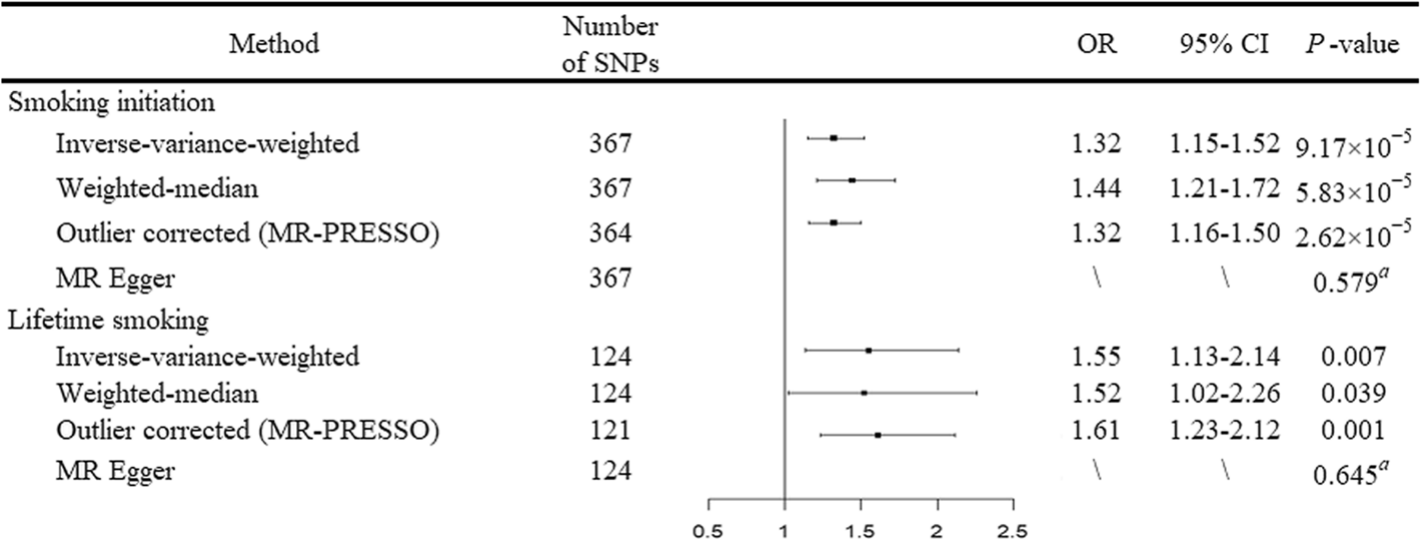
Forest plot of Mendelian randomization analyses for the associations of smoking initiation and lifetime smoking with risk of rheumatoid arthritis. CI, confidence interval; MR, Mendelian randomization; MR-PRESSO test, MR-Pleiotropy RESidual Sum and Outlier test; OR, odds ratio; SNP, single nucleotide polymorphism. ^a^*P* value of the intercept from MR-Egger regression analysis

Supplementary Table 1 presents the 367 SNPs used as IVs for smoking initiation in our MR analysis. Compared with never smokers, genetically predicted smoking initiation was positively associated with an increased risk of RA (odds ratio (OR) = 1.32, 95% confidence interval (CI) = 1.15–1.52, *P* = 9.17 × 10^− 5^) using the IVW method (Fig. 2). Similar effect estimate was obtained using the weighted-median method (OR = 1.44, 95% CI = 1.21–1.72, *P* = 5.83 × 10^− 5^). MR-Egger regression analysis did not suggest evidence of horizontal pleiotropy (*P* intercept = 0.579). Though three possible outlier SNPs were identified using the MR-PRESSO test, the effect estimate of the association between genetically predicted smoking initiation and risk of RA did not change markedly after outlier correction (OR = 1.32, 95% CI = 1.16–1.50, *P* = 2.62 × 10^− 5^).

We then scanned the SNPs used as IVs for their potential secondary phenotypes using the GWAS Catalog. A total of 60 SNPs associated with smoking initiation were found to be associated with other traits (Supplementary Table 2). Excluding these SNPs that are associated with traits other than smoking did not change the results essentially (OR = 1.25, 95% CI = 1.07–1.47, *P* = 0.004 using the IVW method). In the multivariable MR analysis including the SNPs associated with alcohol consumption, education attainment, and BMI, the association between genetically predicted smoking initiation and risk of RA remained in the same pattern (OR = 1.37, 95% CI = 1.19–1.58, *P* = 1.96 × 10^− 5^).

Similarly, we found that genetically predicted lifetime smoking was positively associated with an increased risk of RA (OR = 1.55, 95% CI = 1.13–2.14, *P* = 0.007 for the IVW method). The weighted-median method also produced a consistent effect estimate (OR = 1.52, 95% CI = 1.02–2.26, *P* = 0.039). After correction for outlier SNPs (*n* = 3), the association remained statistically significant (OR = 1.61, 95% CI = 1.23–2.12, *P* = 0.001 in the MR-PRESSO test). Additionally, there was no indication for directional pleiotropy (*P* intercept = 0.645 in the MR-Egger regression).

## Discussion

This is the first MR study to examine whether smoking is causally associated with risk of RA. Our study based on genetics provides evidence that smoking is causally associated with an increased risk of RA. Compared with never smokers, genetic predisposition to smoking was associated with a 32% (95% CI 15%–52%) increased risk of developing RA. Furthermore, genetically predicted lifetime smoking was associated with a 55% (95% CI 13%–114%) increased risk of RA. These findings corroborate the results from the meta-analysis of observational epidemiological studies which showed that self-reported current smokers had a 40% increased risk of RA, compared to never smokers [3].

Although the underlying biological effect of smoking in the development of RA is still unclear [16], there are several plausible explanations. For example, studies have suggested that cigarette smoking can increase oxidative stress in the body through its content of oxidant gasses (e.g., free radicals) and other toxic substances (e.g., nicotine), which may increase the risk of RA through impaired antioxidant systems, platelet activation and inflammation [17]. In addition, chronic smoking can adversely affect the innate and adaptive immune responses and trigger various morphological, physiological, biochemical, and enzymatic changes that lead to impaired antibacterial defenses, cellular regulatory activity, and inflammatory responses, which may contribute to the development of RA [17]. Furthermore, it has been hypothesized that smoking could interact with *HLA-DR shared epitope* genes and trigger HLA-DR-restricted immune reactions to autoantigens modified by citrullination [18]. Though these explanations are biologically plausible, further studies are warranted to elucidate the underlying mechanisms of smoking in the development of RA.

The major strength of this study is that the MR design allows us to investigate the causal nature of the association between smoking and RA. One limitation is that we were unable to stratify the analysis by sex and smoking status, and therefore could not assess gender discrepancies and potential nonlinear associations between smoking and risk of RA. Another limitation is that our analyses were restricted to participants of European ancestry; therefore, our results may not necessarily apply to populations of other ethnicities. However, this fact also reduces the potential bias caused by population stratification.

The validity of the MR approach relies on the following three key assumptions. First, the genetic variants selected as IVs should be strongly associated with the risk factor of interest; second, the genetic variants used as IVs should not be associated with any confounders; and third, the IVs should affect the risk of the outcome merely through the risk factor, not via any alternative pathways [19]. In the current study, we only used SNPs that are strongly associated with smoking initiation and lifetime smoking at genome-wide significance threshold. The *F*-statistics for smoking initiation and lifetime smoking was 79 and 13, respectively, which reduced the chance of weak instrument bias and the possible violation of the first assumption. As genotype is presumed to be randomly assorted at conception, covariates are anticipated to be randomly distributed with respect to genotype. However, it is possible that some SNPs used as IVs are associated with smoking as well as other traits if smoking is causally associated with these secondary phenotypes. It is also possible that some SNPs are associated with multiple pathways, including those not involving smoking. In the current study, we performed the MR-Egger regression and MR-PRESSO tests, which did not indicate the presence of directional pleiotropy. Sensitivity analyses using alternative MR methods including weighted-median and multivariable MR analyses also demonstrated a consistent association. In addition, we manually scanned each of the SNPs used as IVs for potential secondary phenotypes in the GWAS Catalog; MR analyses excluding these SNPs produced similar results.

## Conclusion

In summary, using the MR approach, we have found that genetic predisposition to smoking is associated with risk of RA, suggesting that there is a causal relationship between smoking and the development of RA.

## Supplementary information

**Additional file 1: Table S1.** Effect estimates of the associations between the instrumental variables for smoking initiation and risk of rheumatoid arthritis. Abbreviations: Chr, Chromosome; RA, rheumatoid arthritis; SE, standard error; SNP, single nucleotide polymorphism. **Table S2.** The potential secondary phenotypes of the instrumental variables used for smoking initiation (from the GWAS catalog)^a^. Abbreviations: Chr, chromosome; EA, effect allele; SNP, single nucleotide polymorphism. ^a^Traits associated with the SNP according to previous genome-wide association studies.

## Data Availability

Summary statistics for RA are available at http://plaza.umin.ac.jp/~yokada/datasource/software.htm.

## Abbreviations

CI: Confidence interval
GWAS: Genome-wide association studies
IV: Instrumental variable
IVW: Inverse-variance weighted
MR: Mendelian randomization
MR-PRESSO: MR-Pleiotropy RESidual Sum and Outlier
OR: Odds ratio
RA: Rheumatoid arthritis
SNP: Single nucleotide polymorphism
