# Supplementary material for: Genetic predisposition to smoking is associated with risk of rheumatoid arthritis: a Mendelian randomization study
Source: Arthritis Res Ther. 2020 Mar 6;22:44. doi: 10.1186/s13075-020-2134-1 (PMC7060545; doi:10.1186/s13075-020-2134-1)
Supplement: Supplementary file 1 — Additional file 1: Table S1. Effect estimates of the associations between the instrumental variables for smoking initiation and risk of rheumatoid arthritis. Abbreviations: Chr, Chromosome; RA, rheumatoid arthritis; SE, standard error; SNP, single nucleotide polymorphism. Table S2. The potential secondary phenotypes of the instrumental variables used for smoking initiation (from the GWAS catalog)a. Abbreviations: Chr, chromosome; EA, effect allele; SNP, single nucleotide polymorphism. aTraits associated with the SNP according to previous genome-wide association studies. [file 13075_2020_2134_MOESM1_ESM.docx]

**SUPPLEMENTAL MATERIALS**

**Supplementary Table 1** Effect estimates of the associations between the instrumental variables for smoking initiation and risk of rheumatoid arthritis. Abbreviations: Chr, Chromosome; RA, rheumatoid arthritis; SE, standard error; SNP, single nucleotide polymorphism.

**Supplementary Table 2** The potential secondary phenotypes of the instrumental variables used for smoking initiation (from the GWAS catalog)^a^. Abbreviations: Chr, chromosome; EA, effect allele; SNP, single nucleotide polymorphism. ^a^Traits associated with the SNP according to previous genome-wide association studies.

| **Supplementary Table 1** Effect estimates of the associations between the instrumental variables for smoking initiation and risk of rheumatoid arthritis. | | | | | | | | | | | | |
| --- | --- | --- | --- | --- | --- | --- | --- | --- | --- | --- | --- | --- |
| SNP | Chr | Position | Nearest gene | Effect allele | Non-effect allele | Minor Allele Frequency | Smoking initiation | | | RA | | |
|  |  |  |  |  |  |  | Beta | SE | *P* value | Beta | SE | *P* value |
| rs12130857 | 1 | 7791461 | Intron: CAMTA1 | A | G | 0.325 | -0.018 | 0.003 | 3.65E-11 | 0.010 | 0.018 | 0.44 |
| rs301807 | 1 | 8484823 | Intron: LOC102724552\|RERE | G | A | 0.43 | 0.018 | 0.003 | 2.50E-12 | 0.030 | 0.021 | 0.10 |
| rs3820277 | 1 | 18436657 | Intron: IGSF21 | T | G | 0.474 | -0.019 | 0.003 | 1.57E-13 | 0.020 | 0.020 | 0.37 |
| rs1889571 | 1 | 32195819 | Intron: ADGRB2 | G | T | 0.131 | 0.022 | 0.004 | 4.19E-09 | -0.010 | 0.025 | 0.71 |
| rs10914684 | 1 | 33795572 | Intron: PHC2 | A | G | 0.324 | -0.016 | 0.003 | 6.32E-09 | 0.000 | 0.020 | 0.89 |
| rs2637869 | 1 | 38757237 | Intergenic | A | G | 0.297 | 0.018 | 0.003 | 6.54E-11 | -0.020 | 0.021 | 0.34 |
| rs12755632 | 1 | 41776623 | Intergenic | G | A | 0.316 | -0.015 | 0.003 | 1.93E-08 | 0.010 | 0.021 | 0.56 |
| rs951740 | 1 | 44011737 | Intron: PTPRF | A | G | 0.375 | 0.030 | 0.003 | 3.82E-29 | 0.020 | 0.020 | 0.44 |
| rs925524 | 1 | 46496709 | Synonymous: MAST2 | G | A | 0.29 | 0.016 | 0.003 | 2.94E-08 | 0.062 | 0.021 | 0.01 |
| rs12022778 | 1 | 50603995 | Intron: ELAVL4 | C | A | 0.202 | 0.027 | 0.003 | 3.18E-17 | 0.020 | 0.024 | 0.31 |
| rs11587399 | 1 | 50861071 | Intergenic | T | A | 0.221 | -0.018 | 0.003 | 7.25E-09 | 0.000 | 0.023 | 0.86 |
| rs4912332 | 1 | 58815243 | Intergenic | T | C | 0.491 | 0.014 | 0.003 | 2.94E-08 | 0.020 | 0.020 | 0.39 |
| rs1937443 | 1 | 66469643 | Intron: PDE4B | G | C | 0.437 | 0.020 | 0.003 | 1.79E-15 | 0.020 | 0.020 | 0.41 |
| rs1022528 | 1 | 71490122 | Intron: PTGER3 | A | G | 0.344 | 0.017 | 0.003 | 8.48E-11 | 0.010 | 0.020 | 0.52 |
| rs12740789 | 1 | 72752073 | Intergenic | A | G | 0.178 | -0.028 | 0.003 | 1.18E-17 | -0.030 | 0.026 | 0.22 |
| rs80054503 | 1 | 72900406 | Intergenic | C | T | 0.11 | -0.024 | 0.004 | 3.10E-09 | 0.030 | 0.026 | 0.21 |
| rs10789369 | 1 | 73824909 | Intergenic | G | A | 0.385 | -0.023 | 0.003 | 3.39E-19 | 0.000 | 0.020 | 0.86 |
| rs1514176 | 1 | 74991596 | Intron: FPGT-TNNI3K\|TNNI3K | A | G | 0.42 | -0.019 | 0.003 | 7.67E-14 | -0.030 | 0.018 | 0.20 |
| rs10873871 | 1 | 76689019 | Intron: ST6GALNAC3 | G | A | 0.207 | 0.017 | 0.003 | 2.82E-08 | -0.020 | 0.025 | 0.37 |
| rs11162019 | 1 | 87913176 | Intergenic | T | C | 0.363 | -0.015 | 0.003 | 5.06E-09 | 0.030 | 0.020 | 0.14 |
| rs1008078 | 1 | 91189731 | Intergenic | T | C | 0.402 | 0.023 | 0.003 | 1.63E-18 | -0.020 | 0.016 | 0.25 |
| rs1935571 | 1 | 96414335 | Intergenic | G | T | 0.48 | -0.016 | 0.003 | 6.99E-10 | 0.041 | 0.019 | 0.02 |
| rs12027999 | 1 | 154206358 | Intron: UBAP2L | C | T | 0.12 | -0.024 | 0.004 | 5.33E-10 | 0.000 | 0.028 | 0.93 |
| rs45444697 | 1 | 155034632 | Intron: ADAM15\|DCST1-AS1 | G | C | 0.212 | 0.020 | 0.003 | 2.72E-10 | 0.039 | 0.020 | 0.05 |
| rs2901785 | 1 | 174104743 | Intron: LOC102724601 | A | G | 0.446 | -0.017 | 0.003 | 1.47E-11 | -0.030 | 0.021 | 0.13 |
| rs35656245 | 1 | 190957480 | Intergenic | A | G | 0.276 | 0.016 | 0.003 | 2.23E-08 | 0.020 | 0.022 | 0.34 |
| rs12739243 | 1 | 210302043 | Intron: SYT14 | C | T | 0.221 | -0.021 | 0.003 | 4.45E-12 | 0.020 | 0.024 | 0.29 |
| rs12563365 | 1 | 236872829 | Intron: ACTN2 | A | G | 0.444 | 0.017 | 0.003 | 1.05E-10 | 0.000 | 0.020 | 0.99 |
| rs876793 | 1 | 237852083 | Intron: RYR2 | C | T | 0.349 | -0.018 | 0.003 | 5.69E-11 | -0.010 | 0.023 | 0.78 |
| rs114976176 | 2 | 264621 | Intron: SH3YL1 | C | A | 0.352 | -0.016 | 0.003 | 6.04E-09 | -0.010 | 0.020 | 0.61 |
| rs6731872 | 2 | 624205 | Intergenic | G | T | 0.174 | 0.032 | 0.003 | 5.35E-21 | -0.020 | 0.025 | 0.45 |
| rs1022376 | 2 | 22067213 | Intergenic | C | T | 0.484 | -0.015 | 0.003 | 1.66E-08 | 0.000 | 0.020 | 0.96 |
| rs61533748 | 2 | 22582968 | Intergenic | C | T | 0.384 | 0.017 | 0.003 | 2.82E-11 | 0.000 | 0.020 | 0.87 |
| rs72790288 | 2 | 29513404 | Intron: ALK | A | G | 0.0282 | -0.046 | 0.008 | 3.28E-09 | -0.010 | 0.056 | 0.90 |
| rs2710634 | 2 | 32808804 | Intron: BIRC6 | C | T | 0.479 | -0.018 | 0.003 | 3.36E-12 | -0.030 | 0.020 | 0.21 |
| rs62137126 | 2 | 44250149 | Intergenic | G | A | 0.121 | -0.024 | 0.004 | 1.31E-09 | -0.010 | 0.030 | 0.86 |
| rs1004787 | 2 | 45159091 | Intron: LINC01833 | A | G | 0.448 | 0.028 | 0.003 | 1.11E-28 | 0.039 | 0.020 | 0.07 |
| rs7598402 | 2 | 50735943 | Intron: NRXN1 | G | C | 0.492 | -0.015 | 0.003 | 7.38E-09 | 0.000 | 0.018 | 0.80 |
| rs10490159 | 2 | 51341259 | Intron: LOC730100 | T | C | 0.394 | 0.017 | 0.003 | 3.86E-11 | -0.020 | 0.021 | 0.34 |
| rs1518393 | 2 | 58171220 | Intron: VRK2 | C | A | 0.381 | 0.017 | 0.003 | 1.30E-10 | 0.010 | 0.015 | 0.47 |
| rs17616642 | 2 | 59022210 | Intron: LINC01122 | G | A | 0.247 | -0.017 | 0.003 | 2.10E-08 | -0.030 | 0.020 | 0.11 |
| rs6730325 | 2 | 59315828 | Intergenic | A | G | 0.39 | -0.015 | 0.003 | 2.10E-08 | -0.020 | 0.021 | 0.32 |
| rs2539706 | 2 | 59819545 | Intergenic | A | G | 0.47 | 0.016 | 0.003 | 1.95E-10 | 0.020 | 0.015 | 0.16 |
| rs7585579 | 2 | 60024857 | Intergenic | G | C | 0.499 | 0.020 | 0.003 | 5.48E-15 | 0.030 | 0.017 | 0.09 |
| rs1863161 | 2 | 60139524 | Intergenic | A | G | 0.439 | 0.015 | 0.003 | 2.34E-09 | -0.020 | 0.018 | 0.21 |
| rs359247 | 2 | 60477052 | Intergenic | T | A | 0.361 | 0.022 | 0.003 | 9.89E-17 | 0.000 | 0.020 | 0.99 |
| rs62180324 | 2 | 63416606 | Intron: WDPCP | A | G | 0.212 | -0.020 | 0.003 | 3.91E-10 | 0.039 | 0.017 | 0.02 |
| rs6750107 | 2 | 80748807 | Intron: CTNNA2 | A | G | 0.387 | 0.015 | 0.003 | 2.60E-08 | -0.020 | 0.018 | 0.40 |
| rs12714017 | 2 | 80999398 | Intergenic | C | T | 0.489 | 0.015 | 0.003 | 3.65E-09 | -0.020 | 0.020 | 0.37 |
| rs56208390 | 2 | 83247997 | Intergenic | G | A | 0.123 | 0.022 | 0.004 | 2.68E-08 | 0.030 | 0.029 | 0.39 |
| rs11692435 | 2 | 98275354 | Nonsynonymous: ACTR1B | A | G | 0.0848 | 0.025 | 0.005 | 4.47E-08 | -0.051 | 0.040 | 0.24 |
| rs13392222 | 2 | 100672408 | Intron: AFF3 | C | A | 0.139 | -0.023 | 0.004 | 1.93E-10 | -0.030 | 0.022 | 0.13 |
| rs1901477 | 2 | 104126983 | Intergenic | G | A | 0.489 | 0.030 | 0.003 | 2.07E-31 | 0.010 | 0.018 | 0.46 |
| rs11889814 | 2 | 104432494 | Intergenic | C | A | 0.128 | -0.021 | 0.004 | 3.44E-08 | -0.039 | 0.029 | 0.24 |
| rs3811038 | 2 | 113240183 | Intron: TTL | C | T | 0.279 | 0.019 | 0.003 | 1.58E-11 | -0.010 | 0.020 | 0.71 |
| rs75210106 | 2 | 113246436 | Intron: TTL | T | C | 0.177 | -0.019 | 0.003 | 2.33E-08 | 0.020 | 0.026 | 0.34 |
| rs34399632 | 2 | 137571174 | Intron: THSD7B | G | A | 0.232 | 0.019 | 0.003 | 1.46E-10 | -0.010 | 0.025 | 0.68 |
| rs74697736 | 2 | 145412271 | Intergenic | A | G | 0.287 | 0.022 | 0.003 | 2.43E-15 | 0.058 | 0.022 | 0.01 |
| rs6756212 | 2 | 146140132 | Intergenic | T | C | 0.465 | -0.034 | 0.003 | 3.49E-40 | -0.020 | 0.021 | 0.32 |
| rs16826827 | 2 | 147825689 | Intergenic | C | T | 0.124 | -0.022 | 0.004 | 9.17E-09 | 0.000 | 0.031 | 0.99 |
| rs1445649 | 2 | 155682556 | Intron: KCNJ3 | C | T | 0.462 | 0.021 | 0.003 | 8.48E-16 | -0.010 | 0.020 | 0.76 |
| rs1722666 | 2 | 161816880 | Intergenic | T | C | 0.268 | 0.016 | 0.003 | 2.17E-08 | -0.030 | 0.018 | 0.11 |
| rs11678980 | 2 | 162101261 | Exon: LINC01806 | A | G | 0.45 | 0.018 | 0.003 | 5.19E-12 | 0.000 | 0.020 | 0.97 |
| rs12474587 | 2 | 162802993 | Intron: SLC4A10 | T | G | 0.429 | 0.024 | 0.003 | 4.83E-21 | 0.000 | 0.015 | 1.00 |
| rs357304 | 2 | 164862639 | Intergenic | C | T | 0.273 | 0.017 | 0.003 | 5.40E-09 | 0.000 | 0.020 | 0.94 |
| rs13007361 | 2 | 166250244 | Intergenic | A | G | 0.208 | 0.018 | 0.003 | 2.29E-08 | 0.000 | 0.023 | 0.84 |
| rs7600835 | 2 | 172521827 | Intergenic | A | G | 0.342 | -0.015 | 0.003 | 1.80E-08 | -0.010 | 0.023 | 0.69 |
| rs6750529 | 2 | 182027603 | Intron: LINC01934 | T | C | 0.256 | 0.020 | 0.003 | 9.26E-12 | 0.010 | 0.018 | 0.56 |
| rs17229285 | 2 | 199523122 | Intergenic | T | C | 0.495 | -0.015 | 0.003 | 1.27E-09 | -0.030 | 0.016 | 0.05 |
| rs3115418 | 2 | 200936399 | Intergenic | C | T | 0.454 | -0.014 | 0.003 | 2.79E-08 | 0.010 | 0.021 | 0.57 |
| rs62193862 | 2 | 202843875 | Intergenic | A | G | 0.0999 | 0.024 | 0.004 | 1.99E-08 | -0.020 | 0.042 | 0.58 |
| rs4674916 | 2 | 225365635 | Intron: CUL3 | A | C | 0.328 | -0.018 | 0.003 | 3.06E-11 | -0.030 | 0.021 | 0.12 |
| rs4674993 | 2 | 226332033 | Intron: NYAP2 | G | A | 0.2 | -0.024 | 0.003 | 4.85E-14 | -0.039 | 0.020 | 0.08 |
| rs11713899 | 3 | 2365026 | Intron: CNTN4 | C | A | 0.171 | 0.019 | 0.003 | 3.15E-08 | 0.010 | 0.026 | 0.71 |
| rs748832 | 3 | 16851202 | Intergenic | G | A | 0.371 | 0.017 | 0.003 | 6.60E-11 | 0.041 | 0.019 | 0.03 |
| rs10446419 | 3 | 25725501 | Intergenic | G | A | 0.207 | -0.020 | 0.003 | 5.05E-10 | 0.020 | 0.026 | 0.47 |
| rs13319205 | 3 | 47800216 | Intron: SMARCC1 | A | T | 0.29 | 0.017 | 0.003 | 3.77E-09 | 0.020 | 0.020 | 0.37 |
| rs3172494 | 3 | 48731487 | Utr3: IP6K2 | T | G | 0.115 | -0.029 | 0.004 | 3.40E-13 | 0.000 | 0.026 | 0.96 |
| rs2526390 | 3 | 50192760 | Intron: SEMA3F\|SEMA3F-AS1 | T | C | 0.334 | 0.020 | 0.003 | 3.62E-14 | -0.010 | 0.015 | 0.53 |
| rs2276825 | 3 | 52886605 | Intron: STIMATE\|TMEM110-MUSTN1 | C | T | 0.245 | 0.019 | 0.003 | 1.89E-10 | 0.020 | 0.018 | 0.22 |
| rs2306866 | 3 | 53766212 | Intron: CACNA1D | T | A | 0.386 | -0.017 | 0.003 | 1.89E-10 | 0.000 | 0.018 | 0.76 |
| rs73831818 | 3 | 55988394 | Intron: ERC2 | G | A | 0.057 | 0.032 | 0.005 | 5.46E-09 | 0.030 | 0.042 | 0.39 |
| rs1910236 | 3 | 59434420 | Intergenic | A | G | 0.469 | 0.015 | 0.003 | 9.91E-09 | 0.049 | 0.019 | 0.01 |
| rs7640107 | 3 | 59966156 | Intron: FHIT | T | C | 0.431 | -0.014 | 0.003 | 3.46E-08 | -0.010 | 0.021 | 0.60 |
| rs2734390 | 3 | 60459291 | Intron: FHIT | G | A | 0.372 | 0.015 | 0.003 | 2.09E-08 | 0.010 | 0.015 | 0.66 |
| rs221988 | 3 | 64234307 | Intergenic | C | A | 0.384 | -0.015 | 0.003 | 1.43E-08 | -0.010 | 0.020 | 0.75 |
| rs2196356 | 3 | 70890288 | Intergenic | C | G | 0.289 | -0.019 | 0.003 | 2.45E-11 | 0.030 | 0.021 | 0.14 |
| rs11128203 | 3 | 71064431 | Intron: FOXP1 | A | T | 0.47 | 0.020 | 0.003 | 1.29E-15 | 0.020 | 0.020 | 0.42 |
| rs62246017 | 3 | 71483084 | Intron: FOXP1 | A | G | 0.323 | -0.016 | 0.003 | 3.03E-09 | -0.020 | 0.023 | 0.46 |
| rs4543050 | 3 | 74954560 | Intergenic | T | A | 0.184 | 0.022 | 0.003 | 1.45E-11 | 0.020 | 0.026 | 0.37 |
| rs6782116 | 3 | 77176032 | Intron: ROBO2 | T | C | 0.415 | -0.015 | 0.003 | 1.46E-08 | 0.030 | 0.020 | 0.19 |
| rs13066050 | 3 | 81325861 | Intergenic | T | C | 0.208 | 0.019 | 0.003 | 1.93E-09 | -0.020 | 0.023 | 0.45 |
| rs12633090 | 3 | 83241365 | Intergenic | C | G | 0.182 | -0.023 | 0.003 | 3.16E-12 | -0.049 | 0.024 | 0.06 |
| rs1549979 | 3 | 85460131 | Intron: CADM2 | T | C | 0.385 | -0.025 | 0.003 | 8.80E-21 | -0.020 | 0.018 | 0.43 |
| rs74664784 | 3 | 85475292 | Intron: CADM2 | C | T | 0.369 | -0.020 | 0.003 | 9.34E-13 | 0.020 | 0.018 | 0.43 |
| rs57153235 | 3 | 85902536 | Intron: CADM2 | G | T | 0.318 | -0.019 | 0.003 | 1.56E-12 | -0.010 | 0.018 | 0.76 |
| rs6437769 | 3 | 107997514 | Intergenic | T | C | 0.419 | 0.014 | 0.003 | 3.74E-08 | -0.010 | 0.021 | 0.51 |
| rs9288999 | 3 | 114147927 | Intron: ZBTB20 | A | G | 0.265 | 0.017 | 0.003 | 1.50E-09 | 0.000 | 0.023 | 0.86 |
| rs6438436 | 3 | 117822149 | Intergenic | T | C | 0.184 | 0.025 | 0.003 | 5.33E-14 | 0.030 | 0.025 | 0.29 |
| rs12053870 | 3 | 118302515 | Intron: LOC105374060 | G | T | 0.458 | 0.016 | 0.003 | 1.02E-09 | -0.020 | 0.020 | 0.44 |
| rs9826984 | 3 | 131945722 | Intergenic | A | G | 0.458 | -0.014 | 0.003 | 3.87E-08 | -0.010 | 0.021 | 0.58 |
| rs2279829 | 3 | 147106319 | Utr3: ZIC4 | T | C | 0.216 | -0.017 | 0.003 | 2.05E-08 | 0.020 | 0.020 | 0.30 |
| rs2319545 | 3 | 147719648 | Intergenic | A | C | 0.149 | 0.023 | 0.004 | 8.30E-11 | -0.030 | 0.021 | 0.16 |
| rs10935779 | 3 | 149543102 | Intron: RNF13 | T | C | 0.415 | -0.014 | 0.003 | 2.95E-08 | -0.020 | 0.021 | 0.34 |
| rs963354 | 3 | 157393770 | Intergenic | A | C | 0.313 | 0.015 | 0.003 | 4.21E-08 | 0.000 | 0.020 | 0.91 |
| rs1714521 | 3 | 158284861 | Intron: LOC100996447 | C | A | 0.411 | -0.016 | 0.003 | 3.07E-10 | -0.010 | 0.018 | 0.45 |
| rs1449012 | 3 | 159048333 | Intron: IQCJ-SCHIP1\|SCHIP1 | T | C | 0.463 | -0.015 | 0.003 | 1.77E-09 | -0.020 | 0.018 | 0.42 |
| rs9850597 | 3 | 161761866 | Intergenic | A | G | 0.184 | -0.019 | 0.003 | 1.65E-08 | 0.000 | 0.020 | 0.97 |
| rs1187820 | 3 | 173072584 | Intergenic | T | C | 0.439 | -0.014 | 0.003 | 2.69E-08 | 0.000 | 0.023 | 0.82 |
| rs16828799 | 3 | 173353739 | Intron: NLGN1 | T | G | 0.156 | 0.020 | 0.004 | 1.83E-08 | 0.010 | 0.025 | 0.76 |
| rs9841807 | 3 | 175718927 | Intergenic | T | C | 0.273 | 0.016 | 0.003 | 1.35E-08 | 0.010 | 0.020 | 0.69 |
| rs7631379 | 3 | 181409057 | Intron: SOX2-OT | C | T | 0.206 | 0.021 | 0.003 | 3.94E-11 | -0.030 | 0.030 | 0.31 |
| rs4140932 | 4 | 15458598 | Intergenic | A | T | 0.431 | -0.014 | 0.003 | 4.89E-08 | 0.000 | 0.020 | 0.99 |
| rs12642744 | 4 | 28027176 | Intergenic | T | G | 0.256 | -0.017 | 0.003 | 2.82E-08 | -0.010 | 0.023 | 0.51 |
| rs59537158 | 4 | 28246049 | Intergenic | T | C | 0.214 | 0.022 | 0.003 | 4.62E-13 | 0.068 | 0.026 | 0.00 |
| rs1389171 | 4 | 28822284 | Intergenic | A | T | 0.241 | -0.017 | 0.003 | 4.45E-09 | 0.020 | 0.022 | 0.33 |
| rs55944129 | 4 | 29082156 | Intergenic | C | T | 0.267 | -0.018 | 0.003 | 1.06E-09 | -0.010 | 0.023 | 0.50 |
| rs58400863 | 4 | 31184484 | Intron: LINC02497 | A | G | 0.347 | -0.020 | 0.003 | 4.89E-14 | 0.010 | 0.023 | 0.61 |
| rs7657022 | 4 | 35501032 | Intergenic | G | A | 0.489 | 0.018 | 0.003 | 7.34E-13 | -0.020 | 0.020 | 0.35 |
| rs55900829 | 4 | 35514712 | Intergenic | T | A | 0.328 | 0.019 | 0.003 | 5.63E-12 | 0.020 | 0.020 | 0.35 |
| rs112725451 | 4 | 68017710 | Intergenic | T | C | 0.169 | 0.026 | 0.003 | 1.65E-14 | 0.010 | 0.026 | 0.73 |
| rs1160685 | 4 | 94052854 | Intron: GRID2 | G | C | 0.45 | 0.015 | 0.003 | 2.31E-09 | -0.010 | 0.021 | 0.61 |
| rs1435479 | 4 | 94550450 | Intron: GRID2 | T | G | 0.287 | 0.016 | 0.003 | 5.68E-09 | 0.020 | 0.022 | 0.26 |
| rs3934797 | 4 | 112467612 | Intergenic | A | G | 0.182 | -0.021 | 0.003 | 1.12E-10 | 0.020 | 0.027 | 0.39 |
| rs71602617 | 4 | 136406155 | Intergenic | T | C | 0.216 | -0.018 | 0.003 | 2.10E-08 | -0.010 | 0.026 | 0.62 |
| rs7696257 | 4 | 137474783 | Intergenic | A | G | 0.366 | 0.015 | 0.003 | 6.78E-09 | 0.049 | 0.022 | 0.03 |
| rs13109980 | 4 | 140886963 | Intron: MAML3 | A | G | 0.326 | -0.022 | 0.003 | 3.37E-16 | 0.010 | 0.020 | 0.72 |
| rs1116690 | 4 | 143510148 | Intron: INPP4B | G | A | 0.258 | 0.016 | 0.003 | 2.16E-08 | -0.068 | 0.021 | 0.00 |
| rs13110073 | 4 | 147797913 | Intron: TTC29 | C | T | 0.395 | -0.025 | 0.003 | 3.24E-21 | 0.010 | 0.018 | 0.80 |
| rs28717373 | 4 | 147985231 | Intergenic | T | C | 0.356 | -0.016 | 0.003 | 6.16E-10 | 0.000 | 0.020 | 0.82 |
| rs62340589 | 4 | 176875795 | Intron: GPM6A | C | G | 0.201 | 0.017 | 0.003 | 4.31E-08 | 0.010 | 0.026 | 0.60 |
| rs12517438 | 5 | 30842054 | Intergenic | G | T | 0.462 | 0.015 | 0.003 | 1.89E-09 | -0.010 | 0.018 | 0.77 |
| rs35375873 | 5 | 43190647 | Intergenic | C | G | 0.11 | -0.027 | 0.004 | 3.29E-11 | 0.000 | 0.059 | 1.00 |
| rs986714 | 5 | 50821338 | Intergenic | T | A | 0.445 | -0.016 | 0.003 | 4.13E-10 | -0.030 | 0.020 | 0.16 |
| rs71592686 | 5 | 60121271 | Intron: ELOVL7 | C | T | 0.274 | 0.021 | 0.003 | 3.85E-13 | -0.020 | 0.017 | 0.26 |
| rs2028269 | 5 | 79308315 | Intron: THBS4 | A | G | 0.399 | 0.016 | 0.003 | 5.19E-10 | 0.000 | 0.020 | 0.99 |
| rs6874731 | 5 | 80263865 | Intron: RASGRF2 | G | T | 0.484 | 0.015 | 0.003 | 1.83E-09 | -0.049 | 0.019 | 0.01 |
| rs6452785 | 5 | 87685500 | Intron: TMEM161B-AS1 | T | C | 0.474 | -0.027 | 0.003 | 4.69E-26 | 0.020 | 0.020 | 0.41 |
| rs10805858 | 5 | 88873832 | Intergenic | T | A | 0.335 | 0.018 | 0.003 | 1.88E-11 | 0.000 | 0.023 | 0.84 |
| rs42417 | 5 | 94198290 | Intron: MCTP1 | T | C | 0.309 | 0.017 | 0.003 | 8.27E-10 | 0.030 | 0.022 | 0.13 |
| rs72780746 | 5 | 103929588 | Intergenic | C | T | 0.173 | -0.026 | 0.003 | 2.05E-14 | -0.020 | 0.025 | 0.49 |
| rs10060196 | 5 | 106455988 | Intergenic | A | C | 0.419 | 0.018 | 0.003 | 1.29E-12 | 0.010 | 0.015 | 0.67 |
| rs72789626 | 5 | 106825618 | Intron: EFNA5 | A | T | 0.136 | -0.026 | 0.004 | 5.13E-12 | 0.039 | 0.027 | 0.22 |
| rs17165769 | 5 | 107365642 | Intron: FBXL17 | G | A | 0.395 | 0.016 | 0.003 | 9.56E-10 | -0.020 | 0.020 | 0.23 |
| rs329124 | 5 | 133865452 | Intron: JADE2 | G | A | 0.428 | -0.016 | 0.003 | 1.96E-10 | -0.049 | 0.015 | 0.00 |
| rs1385108 | 5 | 154839646 | Intergenic | T | C | 0.239 | 0.019 | 0.003 | 3.84E-10 | 0.030 | 0.017 | 0.09 |
| rs1173461 | 5 | 157707571 | Intergenic | T | C | 0.327 | 0.017 | 0.003 | 9.51E-10 | -0.020 | 0.021 | 0.28 |
| rs11956866 | 5 | 161018271 | Intergenic | G | T | 0.433 | -0.015 | 0.003 | 7.82E-09 | -0.010 | 0.015 | 0.40 |
| rs3909281 | 5 | 165096435 | Intergenic | G | T | 0.464 | 0.021 | 0.003 | 1.62E-16 | 0.020 | 0.021 | 0.22 |
| rs3843905 | 5 | 165427280 | Intergenic | T | C | 0.403 | -0.015 | 0.003 | 5.41E-09 | -0.010 | 0.018 | 0.75 |
| rs6890961 | 5 | 166778503 | Intron: TENM2 | T | C | 0.376 | -0.019 | 0.003 | 2.13E-13 | 0.000 | 0.023 | 0.87 |
| rs4044321 | 5 | 166989513 | Intron: TENM2 | G | A | 0.356 | -0.023 | 0.003 | 1.75E-17 | 0.000 | 0.020 | 0.94 |
| rs2173019 | 5 | 167614971 | Intron: TENM2 | A | T | 0.177 | 0.028 | 0.003 | 2.98E-17 | 0.000 | 0.026 | 0.93 |
| rs10042827 | 5 | 170299916 | Intron: RANBP17 | C | T | 0.319 | 0.017 | 0.003 | 9.41E-10 | 0.020 | 0.021 | 0.30 |
| rs359431 | 5 | 173288534 | Intergenic | T | C | 0.44 | -0.014 | 0.003 | 3.16E-08 | -0.020 | 0.016 | 0.14 |
| rs1059490 | 6 | 26171250 | Utr3: HIST1H2BD | C | T | 0.367 | -0.019 | 0.003 | 2.16E-12 | 0.030 | 0.016 | 0.08 |
| rs6932350 | 6 | 26571629 | Intron: LOC105374988 | A | T | 0.455 | 0.015 | 0.003 | 5.13E-09 | -0.030 | 0.016 | 0.08 |
| rs1150668 | 6 | 28129789 | Intron: ZNF192P1 | G | T | 0.419 | -0.019 | 0.003 | 8.54E-13 | -0.039 | 0.017 | 0.01 |
| rs1632941 | 6 | 29796685 | Intron: HLA-G | C | T | 0.46 | -0.016 | 0.003 | 6.67E-10 | -0.140 | 0.015 | 0.00 |
| rs3218116 | 6 | 41901763 | Intergenic | T | C | 0.256 | -0.020 | 0.003 | 1.05E-11 | 0.020 | 0.020 | 0.39 |
| rs160631 | 6 | 52895230 | Intron: ICK | G | T | 0.269 | -0.017 | 0.003 | 1.87E-09 | 0.041 | 0.021 | 0.06 |
| rs7743165 | 6 | 67521222 | Intergenic | G | T | 0.495 | 0.019 | 0.003 | 4.15E-14 | -0.010 | 0.020 | 0.75 |
| rs79180767 | 6 | 67540984 | Intergenic | T | C | 0.238 | 0.020 | 0.003 | 7.00E-12 | 0.000 | 0.020 | 0.88 |
| rs10945141 | 6 | 69470709 | Intron: ADGRB3 | A | G | 0.263 | 0.018 | 0.003 | 3.59E-10 | 0.000 | 0.020 | 0.92 |
| rs17554906 | 6 | 92226609 | Intergenic | C | G | 0.444 | 0.014 | 0.003 | 3.14E-08 | 0.010 | 0.018 | 0.78 |
| rs619087 | 6 | 94175279 | Intergenic | G | A | 0.422 | 0.014 | 0.003 | 3.10E-08 | -0.020 | 0.017 | 0.22 |
| rs6568832 | 6 | 97702876 | Intron: MIR548H3\|MMS22L | A | G | 0.246 | 0.019 | 0.003 | 1.74E-10 | -0.062 | 0.022 | 0.01 |
| rs12195240 | 6 | 98636905 | Intergenic | A | G | 0.285 | 0.025 | 0.003 | 1.08E-18 | -0.010 | 0.021 | 0.62 |
| rs6936160 | 6 | 100347745 | Intergenic | T | C | 0.302 | 0.020 | 0.003 | 4.20E-13 | -0.010 | 0.021 | 0.58 |
| rs12530388 | 6 | 101329173 | Utr5: ASCC3 | C | A | 0.489 | -0.018 | 0.003 | 5.83E-13 | 0.030 | 0.019 | 0.07 |
| rs3800227 | 6 | 108994161 | Intron: FOXO3 | G | A | 0.258 | 0.017 | 0.003 | 3.64E-09 | 0.000 | 0.023 | 0.94 |
| rs118202 | 6 | 111658371 | Intron: REV3L | T | G | 0.188 | -0.037 | 0.003 | 1.90E-29 | 0.010 | 0.020 | 0.54 |
| rs73008357 | 6 | 156431856 | Intergenic | C | A | 0.121 | -0.022 | 0.004 | 2.44E-08 | 0.030 | 0.029 | 0.35 |
| rs10698713 | 6 | 158882320 | Intron: TULP4 | A | G | 0.0544 | -0.034 | 0.006 | 2.38E-09 | 0.068 | 0.043 | 0.09 |
| rs1737329 | 6 | 163807748 | Intergenic | G | C | 0.258 | 0.017 | 0.003 | 5.08E-09 | -0.041 | 0.021 | 0.06 |
| rs10272990 | 7 | 1703675 | Intergenic | C | T | 0.328 | -0.021 | 0.003 | 1.27E-14 | 0.000 | 0.026 | 0.98 |
| rs6948707 | 7 | 1870794 | Intron: MAD1L1 | G | T | 0.419 | 0.024 | 0.003 | 4.24E-21 | 0.030 | 0.016 | 0.05 |
| rs10259715 | 7 | 3329967 | Intergenic | A | T | 0.198 | -0.019 | 0.003 | 6.42E-09 | -0.010 | 0.020 | 0.78 |
| rs13237637 | 7 | 3503207 | Intron: SDK1 | C | G | 0.485 | -0.024 | 0.003 | 1.54E-20 | 0.000 | 0.018 | 0.86 |
| rs79631993 | 7 | 69432311 | Intron: AUTS2 | C | A | 0.211 | -0.017 | 0.003 | 3.67E-08 | -0.020 | 0.020 | 0.37 |
| rs7809303 | 7 | 69484366 | Intron: AUTS2 | A | G | 0.325 | -0.021 | 0.003 | 3.48E-15 | 0.020 | 0.020 | 0.41 |
| rs7802996 | 7 | 77771983 | Intron: MAGI2 | T | C | 0.166 | -0.021 | 0.003 | 1.06E-09 | -0.020 | 0.029 | 0.56 |
| rs1030015 | 7 | 78139581 | Intron: MAGI2 | T | G | 0.48 | 0.014 | 0.003 | 2.15E-08 | 0.000 | 0.015 | 1.00 |
| rs4727189 | 7 | 88442568 | Intron: ZNF804B | C | T | 0.344 | 0.015 | 0.003 | 3.00E-08 | 0.020 | 0.018 | 0.35 |
| rs76841737 | 7 | 91281409 | Intergenic | G | C | 0.103 | -0.023 | 0.004 | 3.26E-08 | 0.010 | 0.033 | 0.77 |
| rs11768481 | 7 | 96629103 | Intron: DLX6-AS1 | A | C | 0.34 | -0.019 | 0.003 | 5.23E-12 | 0.000 | 0.023 | 0.87 |
| rs1799068 | 7 | 97707069 | Intergenic | T | G | 0.379 | 0.017 | 0.003 | 2.59E-10 | -0.020 | 0.021 | 0.23 |
| rs13437771 | 7 | 99071478 | Intron: ZNF789 | G | A | 0.155 | -0.027 | 0.004 | 1.39E-14 | 0.083 | 0.025 | 0.00 |
| rs11766326 | 7 | 111100585 | Intron: IMMP2L | C | T | 0.494 | -0.018 | 0.003 | 1.79E-11 | -0.020 | 0.020 | 0.36 |
| rs6968380 | 7 | 114940159 | Intergenic | A | G | 0.319 | -0.023 | 0.003 | 1.05E-17 | 0.030 | 0.020 | 0.15 |
| rs10233018 | 7 | 117523709 | Intergenic | G | A | 0.484 | 0.025 | 0.003 | 4.77E-22 | 0.030 | 0.021 | 0.12 |
| rs10953957 | 7 | 121954709 | Intergenic | A | G | 0.386 | 0.014 | 0.003 | 3.66E-08 | -0.010 | 0.021 | 0.73 |
| rs77283305 | 7 | 132593831 | Intron: CHCHD3 | A | G | 0.306 | -0.015 | 0.003 | 3.91E-08 | -0.030 | 0.021 | 0.10 |
| rs10279261 | 7 | 133589846 | Intron: EXOC4 | A | G | 0.382 | -0.019 | 0.003 | 6.05E-13 | -0.030 | 0.018 | 0.18 |
| rs1561112 | 7 | 133840652 | Intron: LRGUK | C | T | 0.413 | -0.015 | 0.003 | 3.84E-09 | 0.000 | 0.018 | 0.84 |
| rs2952251 | 8 | 10143164 | Intron: MSRA | G | A | 0.256 | 0.016 | 0.003 | 4.24E-08 | 0.030 | 0.021 | 0.19 |
| rs4326350 | 8 | 10763655 | Intron: XKR6 | G | C | 0.493 | -0.018 | 0.003 | 5.16E-12 | -0.020 | 0.016 | 0.19 |
| rs11780471 | 8 | 27344719 | Intergenic | A | G | 0.063 | -0.039 | 0.005 | 1.57E-13 | -0.083 | 0.041 | 0.06 |
| rs11783093 | 8 | 27425349 | Intergenic | T | C | 0.158 | -0.047 | 0.003 | 2.07E-41 | -0.062 | 0.027 | 0.03 |
| rs1565735 | 8 | 27426077 | Intergenic | A | T | 0.204 | -0.019 | 0.003 | 1.33E-09 | -0.030 | 0.024 | 0.25 |
| rs7836565 | 8 | 52569449 | Intron: PXDNL | T | C | 0.282 | -0.016 | 0.003 | 4.36E-08 | -0.030 | 0.021 | 0.10 |
| rs13261666 | 8 | 59814666 | Intron: TOX | T | G | 0.483 | -0.020 | 0.003 | 4.36E-15 | -0.051 | 0.019 | 0.00 |
| rs3850736 | 8 | 64912021 | Intron: LOC102724623 | G | C | 0.474 | 0.019 | 0.003 | 6.43E-14 | 0.058 | 0.019 | 0.00 |
| rs2063976 | 8 | 91096366 | Intergenic | T | C | 0.335 | -0.020 | 0.003 | 7.45E-14 | 0.020 | 0.020 | 0.32 |
| rs6993429 | 8 | 92733282 | Intergenic | A | C | 0.453 | -0.019 | 0.003 | 9.87E-14 | -0.010 | 0.018 | 0.44 |
| rs6986430 | 8 | 93048104 | Intron: RUNX1T1 | C | T | 0.222 | -0.024 | 0.003 | 1.99E-15 | -0.030 | 0.022 | 0.28 |
| rs9987376 | 8 | 93190014 | Intergenic | G | T | 0.426 | -0.020 | 0.003 | 2.01E-15 | -0.030 | 0.020 | 0.14 |
| rs290601 | 8 | 115374642 | Intergenic | T | C | 0.274 | 0.016 | 0.003 | 1.14E-08 | 0.010 | 0.020 | 0.62 |
| rs3847244 | 9 | 3025368 | Intergenic | T | C | 0.47 | 0.019 | 0.003 | 2.60E-13 | 0.030 | 0.020 | 0.12 |
| rs11791671 | 9 | 3398679 | Intron: RFX3 | T | C | 0.0673 | 0.028 | 0.005 | 4.24E-08 | 0.068 | 0.038 | 0.07 |
| rs7024924 | 9 | 8282399 | Intergenic | C | T | 0.174 | 0.019 | 0.003 | 1.90E-08 | 0.010 | 0.026 | 0.59 |
| rs6474609 | 9 | 10981069 | Intergenic | A | T | 0.413 | -0.016 | 0.003 | 1.71E-09 | -0.010 | 0.021 | 0.74 |
| rs1931431 | 9 | 11161799 | Intergenic | C | G | 0.478 | 0.018 | 0.003 | 8.56E-13 | 0.010 | 0.021 | 0.64 |
| rs7867822 | 9 | 20676454 | Intron: FOCAD | G | A | 0.327 | -0.015 | 0.003 | 2.76E-08 | -0.030 | 0.022 | 0.11 |
| rs10966092 | 9 | 23831658 | Intron: ELAVL2 | C | T | 0.267 | -0.020 | 0.003 | 1.12E-12 | 0.000 | 0.020 | 1.00 |
| rs10969352 | 9 | 29747488 | Intergenic | A | T | 0.5 | 0.014 | 0.003 | 1.82E-08 | 0.020 | 0.020 | 0.31 |
| rs4877285 | 9 | 81354129 | Intergenic | A | G | 0.332 | -0.018 | 0.003 | 2.10E-11 | 0.010 | 0.020 | 0.74 |
| rs1930371 | 9 | 81444104 | Intergenic | T | C | 0.241 | -0.017 | 0.003 | 7.09E-09 | -0.051 | 0.024 | 0.04 |
| rs2378662 | 9 | 86707289 | Intron: LOC101927575 | A | G | 0.459 | 0.015 | 0.003 | 2.67E-09 | 0.000 | 0.018 | 0.90 |
| rs1927901 | 9 | 120519111 | Intergenic | C | T | 0.447 | -0.014 | 0.003 | 3.10E-08 | -0.020 | 0.020 | 0.42 |
| rs4837631 | 9 | 122061948 | Intron: BRINP1 | T | C | 0.446 | -0.015 | 0.003 | 2.03E-09 | 0.000 | 0.018 | 0.88 |
| rs1759433 | 9 | 128073097 | Intron: GAPVD1 | A | G | 0.48 | 0.015 | 0.003 | 1.69E-09 | -0.010 | 0.021 | 0.64 |
| rs34553878 | 9 | 134334588 | Nonsynonymous: PRRC2B | G | A | 0.111 | 0.025 | 0.004 | 1.17E-09 | -0.039 | 0.037 | 0.28 |
| rs7026534 | 9 | 134907263 | Intron: MED27 | G | T | 0.296 | -0.017 | 0.003 | 2.68E-09 | -0.030 | 0.022 | 0.14 |
| rs10858334 | 9 | 137989785 | Utr3: OLFM1 | G | C | 0.14 | 0.023 | 0.004 | 1.18E-09 | 0.010 | 0.030 | 0.84 |
| rs10905461 | 10 | 8803551 | Intergenic | C | T | 0.252 | -0.016 | 0.003 | 2.36E-08 | 0.000 | 0.020 | 0.97 |
| rs7920501 | 10 | 10043159 | Intergenic | A | T | 0.465 | -0.016 | 0.003 | 1.25E-09 | 0.000 | 0.020 | 0.89 |
| rs1291821 | 10 | 11133823 | Intron: CELF2\|CELF2-AS2 | G | A | 0.466 | 0.014 | 0.003 | 1.39E-08 | 0.000 | 0.020 | 0.89 |
| rs11258417 | 10 | 13533053 | Intron: BEND7 | T | C | 0.391 | -0.015 | 0.003 | 2.71E-08 | 0.000 | 0.020 | 0.96 |
| rs7072776 | 10 | 22032942 | Intergenic | G | A | 0.288 | -0.022 | 0.003 | 5.66E-15 | 0.020 | 0.023 | 0.43 |
| rs2796793 | 10 | 36634124 | Intergenic | A | G | 0.452 | 0.014 | 0.003 | 1.55E-08 | 0.039 | 0.020 | 0.04 |
| rs1733760 | 10 | 56698174 | Intron: PCDH15 | C | T | 0.49 | 0.015 | 0.003 | 6.70E-09 | 0.010 | 0.015 | 0.45 |
| rs7921378 | 10 | 63674885 | Intron: ARID5B | C | G | 0.482 | -0.023 | 0.003 | 6.10E-20 | -0.010 | 0.020 | 0.50 |
| rs7901883 | 10 | 103186838 | Intron: BTRC | A | G | 0.23 | -0.019 | 0.003 | 1.98E-10 | 0.020 | 0.022 | 0.36 |
| rs11594623 | 10 | 103960351 | Intergenic | C | T | 0.234 | 0.027 | 0.003 | 7.45E-20 | 0.010 | 0.023 | 0.71 |
| rs11191269 | 10 | 104120522 | Intron: GBF1 | G | C | 0.193 | 0.018 | 0.003 | 4.61E-08 | -0.020 | 0.026 | 0.44 |
| rs28408682 | 10 | 104403310 | Intergenic | G | A | 0.4 | 0.017 | 0.003 | 1.41E-10 | 0.000 | 0.018 | 0.89 |
| rs12244388 | 10 | 104640052 | Intron: AS3MT\|BORCS7-ASMT | A | G | 0.35 | 0.026 | 0.003 | 4.31E-22 | 0.030 | 0.017 | 0.05 |
| rs111842178 | 10 | 104852121 | Intron: NT5C2 | G | A | 0.211 | 0.022 | 0.003 | 2.24E-12 | 0.030 | 0.016 | 0.02 |
| rs34970111 | 10 | 106078937 | Intron: ITPRIP | T | C | 0.458 | -0.015 | 0.003 | 1.28E-08 | 0.010 | 0.020 | 0.66 |
| rs9787523 | 10 | 106460460 | Intron: SORCS3 | C | T | 0.418 | -0.016 | 0.003 | 1.42E-09 | -0.020 | 0.020 | 0.36 |
| rs11192347 | 10 | 106929313 | Intron: SORCS3 | A | G | 0.104 | -0.026 | 0.004 | 6.15E-10 | 0.000 | 0.031 | 0.91 |
| rs10885480 | 10 | 115378364 | Intron: NRAP | C | T | 0.284 | -0.019 | 0.003 | 3.83E-11 | -0.020 | 0.022 | 0.28 |
| rs4752018 | 10 | 118678712 | Intron: SHTN1 | A | C | 0.231 | 0.019 | 0.003 | 4.42E-10 | -0.030 | 0.024 | 0.13 |
| rs9423279 | 10 | 125680419 | Intergenic | G | C | 0.355 | -0.019 | 0.003 | 3.06E-12 | -0.051 | 0.046 | 0.27 |
| rs6265 | 11 | 27679916 | Nonsynonymous: BDNF | T | C | 0.188 | -0.029 | 0.003 | 2.81E-19 | 0.020 | 0.025 | 0.47 |
| rs4275621 | 11 | 28652996 | Intergenic | G | A | 0.382 | -0.021 | 0.003 | 3.76E-16 | -0.020 | 0.022 | 0.24 |
| rs62618693 | 11 | 32956492 | Nonsynonymous: QSER1 | T | C | 0.0428 | -0.035 | 0.006 | 2.09E-08 | -0.062 | 0.057 | 0.29 |
| rs2939756 | 11 | 41436297 | Intron: LRRC4C | A | G | 0.48 | -0.016 | 0.003 | 7.45E-10 | 0.000 | 0.020 | 0.88 |
| rs1381775 | 11 | 42442826 | Intergenic | C | T | 0.288 | -0.016 | 0.003 | 2.79E-08 | 0.030 | 0.021 | 0.16 |
| rs2959084 | 11 | 46078656 | Intron: PHF21A | A | G | 0.295 | 0.017 | 0.003 | 9.82E-10 | 0.010 | 0.020 | 0.60 |
| rs3740977 | 11 | 46393574 | Intron: DGKZ | C | T | 0.167 | 0.019 | 0.003 | 1.17E-08 | -0.058 | 0.027 | 0.04 |
| rs61886926 | 11 | 64133552 | Intron: RPS6KA4 | T | C | 0.384 | -0.018 | 0.003 | 7.30E-12 | -0.041 | 0.016 | 0.00 |
| rs61884449 | 11 | 64485193 | Intron: NRXN2 | T | C | 0.149 | 0.020 | 0.004 | 2.32E-08 | -0.010 | 0.028 | 0.85 |
| rs644740 | 11 | 65561468 | Intron: OVOL1 | T | C | 0.457 | -0.014 | 0.003 | 3.67E-08 | -0.041 | 0.019 | 0.03 |
| rs7943721 | 11 | 73309393 | Intergenic | A | G | 0.171 | -0.021 | 0.003 | 3.58E-10 | -0.020 | 0.029 | 0.53 |
| rs7929518 | 11 | 85980958 | Intron: EED | G | A | 0.227 | 0.019 | 0.003 | 2.55E-10 | 0.020 | 0.024 | 0.30 |
| rs586699 | 11 | 92289734 | Intron: FAT3 | A | G | 0.457 | -0.015 | 0.003 | 7.29E-09 | 0.000 | 0.020 | 0.92 |
| rs2155646 | 11 | 112912811 | Intron: NCAM1 | C | T | 0.4 | 0.038 | 0.003 | 9.44E-48 | 0.020 | 0.018 | 0.23 |
| rs1713676 | 11 | 113660576 | Intergenic | G | A | 0.477 | -0.017 | 0.003 | 5.38E-11 | 0.010 | 0.021 | 0.49 |
| rs238896 | 11 | 113994505 | Intron: ZBTB16 | A | G | 0.49 | -0.017 | 0.003 | 3.65E-11 | -0.020 | 0.021 | 0.31 |
| rs540860 | 11 | 121530888 | Intergenic | G | A | 0.457 | 0.018 | 0.003 | 5.75E-12 | -0.039 | 0.020 | 0.06 |
| rs1944689 | 11 | 121634334 | Intergenic | T | G | 0.214 | 0.018 | 0.003 | 1.27E-08 | 0.020 | 0.025 | 0.39 |
| rs1834306 | 11 | 122023187 | Intron: MIR100HG | G | A | 0.421 | -0.014 | 0.003 | 1.96E-08 | -0.020 | 0.023 | 0.46 |
| rs1106363 | 11 | 131966264 | Intron: NTM | T | C | 0.345 | 0.017 | 0.003 | 9.20E-11 | 0.010 | 0.020 | 0.61 |
| rs2010921 | 11 | 132098205 | Intron: NTM | A | G | 0.311 | 0.017 | 0.003 | 2.47E-10 | -0.041 | 0.021 | 0.03 |
| rs11057005 | 12 | 16748721 | Intron: LMO3 | G | A | 0.441 | -0.016 | 0.003 | 9.12E-10 | -0.010 | 0.020 | 0.71 |
| rs13906 | 12 | 49952394 | Utr3: MCRS1 | T | C | 0.109 | -0.025 | 0.004 | 1.98E-09 | 0.000 | 0.033 | 0.93 |
| rs4759229 | 12 | 56474480 | Intron: ERBB3 | G | A | 0.344 | 0.016 | 0.003 | 6.53E-09 | 0.051 | 0.016 | 0.00 |
| rs7969559 | 12 | 69655167 | Intron: CPSF6 | G | A | 0.287 | -0.017 | 0.003 | 1.53E-09 | -0.020 | 0.022 | 0.32 |
| rs7134009 | 12 | 75263193 | Intergenic | C | T | 0.287 | -0.016 | 0.003 | 4.30E-08 | -0.010 | 0.023 | 0.58 |
| rs1109480 | 12 | 121083279 | Intron: CABP1 | A | G | 0.384 | -0.017 | 0.003 | 1.84E-10 | 0.010 | 0.020 | 0.76 |
| rs11611651 | 12 | 133380790 | Intron: GOLGA3 | A | G | 0.0868 | 0.027 | 0.005 | 2.05E-09 | 0.010 | 0.038 | 0.78 |
| rs17197663 | 13 | 38172867 | Utr5: POSTN | A | G | 0.125 | -0.022 | 0.004 | 2.06E-08 | -0.010 | 0.028 | 0.81 |
| rs4264267 | 13 | 38359676 | Intron: TRPC4 | T | C | 0.473 | 0.015 | 0.003 | 6.82E-09 | -0.010 | 0.021 | 0.54 |
| rs61959481 | 13 | 55834929 | Intergenic | A | G | 0.21 | -0.020 | 0.003 | 7.95E-11 | 0.010 | 0.023 | 0.61 |
| rs3098272 | 13 | 55931424 | Intergenic | C | A | 0.201 | -0.018 | 0.003 | 2.08E-08 | 0.020 | 0.024 | 0.32 |
| rs9538162 | 13 | 59265043 | Intergenic | C | T | 0.416 | 0.017 | 0.003 | 1.76E-11 | -0.010 | 0.020 | 0.51 |
| rs1413119 | 13 | 59339281 | Intergenic | T | C | 0.396 | -0.015 | 0.003 | 4.77E-09 | -0.010 | 0.021 | 0.69 |
| rs56367474 | 13 | 59454139 | Intergenic | T | C | 0.304 | -0.017 | 0.003 | 4.20E-10 | 0.000 | 0.023 | 0.87 |
| rs55786907 | 13 | 59871584 | Intergenic | G | A | 0.162 | 0.019 | 0.003 | 1.84E-08 | -0.020 | 0.022 | 0.29 |
| rs4886207 | 13 | 60705792 | Intron: DIAPH3 | C | T | 0.363 | -0.016 | 0.003 | 8.78E-10 | 0.030 | 0.021 | 0.14 |
| rs9540731 | 13 | 66949370 | Intron: PCDH9 | T | C | 0.491 | -0.018 | 0.003 | 3.42E-12 | -0.051 | 0.019 | 0.00 |
| rs9545155 | 13 | 80191873 | Intergenic | C | T | 0.478 | -0.016 | 0.003 | 3.04E-10 | -0.010 | 0.015 | 0.44 |
| rs1772572 | 13 | 81191176 | Intergenic | A | C | 0.324 | -0.017 | 0.003 | 5.62E-10 | 0.020 | 0.020 | 0.30 |
| rs75674569 | 13 | 96823724 | Intron: HS6ST3 | A | G | 0.0997 | -0.025 | 0.004 | 2.58E-09 | 0.058 | 0.034 | 0.10 |
| rs7333559 | 13 | 100546450 | Intron: CLYBL\|LOC101927437 | A | G | 0.217 | -0.023 | 0.003 | 5.94E-14 | 0.000 | 0.028 | 0.87 |
| rs1108130 | 13 | 100648356 | Exon: LINC00554 | A | T | 0.212 | 0.024 | 0.003 | 1.57E-14 | 0.030 | 0.027 | 0.33 |
| rs12855717 | 13 | 101252635 | Intergenic | T | C | 0.462 | 0.016 | 0.003 | 1.22E-09 | 0.010 | 0.020 | 0.57 |
| rs12878369 | 14 | 28346502 | Intergenic | A | C | 0.415 | 0.017 | 0.003 | 1.60E-11 | -0.020 | 0.021 | 0.25 |
| rs2145451 | 14 | 29316842 | Intergenic | C | T | 0.193 | -0.020 | 0.003 | 5.44E-10 | -0.010 | 0.028 | 0.61 |
| rs9323328 | 14 | 58653514 | Intergenic | G | A | 0.463 | -0.014 | 0.003 | 2.55E-08 | 0.010 | 0.021 | 0.59 |
| rs1811739 | 14 | 77529375 | Intron: LINC02288 | A | G | 0.248 | 0.018 | 0.003 | 5.97E-10 | 0.039 | 0.022 | 0.06 |
| rs8005334 | 14 | 79563654 | Intron: NRXN3 | G | T | 0.36 | 0.017 | 0.003 | 3.44E-10 | 0.000 | 0.020 | 0.87 |
| rs34940743 | 14 | 80102233 | Intron: NRXN3 | G | A | 0.346 | 0.016 | 0.003 | 2.80E-09 | 0.000 | 0.026 | 0.96 |
| rs2925128 | 14 | 98362355 | Intergenic | T | C | 0.385 | 0.017 | 0.003 | 3.67E-10 | 0.030 | 0.015 | 0.05 |
| rs1381287 | 14 | 98597552 | Intergenic | T | C | 0.467 | 0.018 | 0.003 | 1.81E-12 | -0.041 | 0.016 | 0.01 |
| rs55913542 | 14 | 99693843 | Intron: BCL11B | T | G | 0.175 | 0.019 | 0.003 | 3.25E-08 | 0.039 | 0.025 | 0.17 |
| rs1435672 | 15 | 36399479 | Intergenic | C | T | 0.44 | 0.014 | 0.003 | 3.82E-08 | 0.000 | 0.020 | 0.96 |
| rs281296 | 15 | 47685010 | Intron: SEMA6D | A | G | 0.357 | 0.025 | 0.003 | 1.59E-20 | 0.000 | 0.020 | 0.96 |
| rs1435741 | 15 | 47935843 | Intron: SEMA6D | A | G | 0.433 | 0.018 | 0.003 | 1.09E-12 | 0.039 | 0.020 | 0.03 |
| rs56902655 | 15 | 63898709 | Intergenic | G | T | 0.136 | -0.022 | 0.004 | 4.09E-09 | -0.039 | 0.029 | 0.18 |
| rs2289791 | 15 | 67476952 | Intron: SMAD3 | T | G | 0.247 | -0.018 | 0.003 | 2.01E-09 | -0.051 | 0.019 | 0.01 |
| rs60833441 | 15 | 74048768 | Intergenic | G | A | 0.461 | -0.014 | 0.003 | 2.28E-08 | -0.020 | 0.020 | 0.31 |
| rs62007780 | 15 | 78025464 | Intron: LINGO1 | T | G | 0.416 | -0.016 | 0.003 | 7.48E-10 | 0.000 | 0.020 | 0.84 |
| rs12442563 | 15 | 83893243 | Intergenic | T | G | 0.223 | -0.023 | 0.003 | 3.13E-14 | 0.020 | 0.023 | 0.47 |
| rs4310804 | 15 | 96858409 | Intron: NR2F2-AS1 | G | C | 0.247 | -0.018 | 0.003 | 7.55E-10 | 0.010 | 0.023 | 0.54 |
| rs8027457 | 15 | 99204101 | Intron: IGF1R | C | T | 0.489 | 0.015 | 0.003 | 1.88E-09 | 0.000 | 0.015 | 0.76 |
| rs1139897 | 16 | 720986 | Nonsynonymous: RHOT2 | A | G | 0.23 | -0.024 | 0.003 | 1.77E-15 | 0.000 | 0.023 | 0.85 |
| rs11076962 | 16 | 5811367 | Intergenic | C | T | 0.279 | 0.018 | 0.003 | 1.20E-10 | 0.010 | 0.015 | 0.58 |
| rs7192140 | 16 | 10173748 | Intron: GRIN2A | C | T | 0.498 | -0.017 | 0.003 | 3.40E-11 | -0.030 | 0.020 | 0.17 |
| rs9922607 | 16 | 17570220 | Intergenic | T | C | 0.2 | -0.022 | 0.003 | 3.42E-12 | -0.020 | 0.023 | 0.44 |
| rs9941217 | 16 | 18050926 | Intergenic | G | C | 0.352 | -0.019 | 0.003 | 3.50E-12 | -0.020 | 0.021 | 0.29 |
| rs7188873 | 16 | 24727064 | Intron: TNRC6A | G | A | 0.387 | 0.020 | 0.003 | 8.46E-15 | 0.020 | 0.021 | 0.25 |
| rs6497840 | 16 | 25351633 | Intergenic | A | G | 0.293 | 0.023 | 0.003 | 2.01E-15 | 0.010 | 0.020 | 0.71 |
| rs4785187 | 16 | 49766772 | Intron: ZNF423 | A | G | 0.223 | 0.020 | 0.003 | 6.55E-11 | -0.030 | 0.024 | 0.26 |
| rs8050598 | 16 | 49891964 | Intergenic | T | C | 0.254 | 0.019 | 0.003 | 1.76E-10 | 0.020 | 0.025 | 0.49 |
| rs12918191 | 16 | 50945156 | Intergenic | G | A | 0.243 | -0.020 | 0.003 | 3.14E-11 | -0.020 | 0.020 | 0.25 |
| rs9302604 | 16 | 69576894 | Intergenic | G | A | 0.435 | 0.019 | 0.003 | 3.29E-13 | -0.010 | 0.020 | 0.55 |
| rs9936784 | 16 | 72230694 | Intergenic | G | T | 0.466 | 0.014 | 0.003 | 4.33E-08 | -0.020 | 0.020 | 0.25 |
| rs62052916 | 16 | 72574550 | Intron: LINC01572 | T | A | 0.0701 | -0.032 | 0.005 | 1.62E-10 | 0.062 | 0.038 | 0.13 |
| rs4788676 | 16 | 72950468 | Intron: ZFHX3 | C | T | 0.229 | -0.018 | 0.003 | 4.92E-09 | 0.010 | 0.023 | 0.52 |
| rs61537885 | 16 | 75620118 | Intergenic | C | T | 0.0354 | -0.040 | 0.007 | 8.06E-09 | 0.020 | 0.037 | 0.57 |
| rs117657830 | 16 | 75766873 | Intergenic | G | A | 0.0417 | -0.038 | 0.006 | 3.18E-09 | -0.020 | 0.047 | 0.68 |
| rs1050847 | 16 | 87443734 | Utr3: ZCCHC14 | T | C | 0.441 | -0.015 | 0.003 | 7.37E-09 | -0.020 | 0.021 | 0.33 |
| rs11642231 | 16 | 89608702 | Intron: SPG7 | A | G | 0.369 | -0.016 | 0.003 | 3.44E-09 | -0.051 | 0.019 | 0.02 |
| rs4790874 | 17 | 1995177 | Intron: SMG6 | T | C | 0.468 | 0.017 | 0.003 | 8.43E-12 | 0.000 | 0.020 | 0.88 |
| rs11078713 | 17 | 7795972 | Intron: CHD3 | G | A | 0.419 | -0.015 | 0.003 | 1.59E-08 | 0.010 | 0.021 | 0.51 |
| rs28441558 | 17 | 7803118 | Intron: CHD3 | C | T | 0.0563 | -0.036 | 0.006 | 1.24E-10 | -0.010 | 0.043 | 0.78 |
| rs11651955 | 17 | 16235462 | Intergenic | A | G | 0.499 | -0.014 | 0.003 | 3.74E-08 | -0.051 | 0.021 | 0.01 |
| rs67777803 | 17 | 27323322 | Intron: SEZ6 | T | G | 0.172 | -0.025 | 0.003 | 3.18E-13 | 0.000 | 0.026 | 0.93 |
| rs2344976 | 17 | 30685935 | Intron: ZNF207 | C | T | 0.388 | -0.015 | 0.003 | 7.98E-09 | 0.051 | 0.019 | 0.02 |
| rs3764351 | 17 | 37824339 | Intron: PNMT | A | G | 0.343 | -0.015 | 0.003 | 3.89E-08 | 0.068 | 0.017 | 0.00 |
| rs72836318 | 17 | 44121579 | Intron: KANSL1 | C | T | 0.246 | -0.017 | 0.003 | 7.00E-09 | 0.020 | 0.018 | 0.34 |
| rs17692129 | 17 | 44793283 | Intron: NSF | T | C | 0.331 | 0.020 | 0.003 | 4.57E-13 | 0.020 | 0.023 | 0.43 |
| rs75919030 | 17 | 50193197 | Intron: CA10 | C | T | 0.267 | -0.021 | 0.003 | 3.35E-13 | -0.010 | 0.023 | 0.64 |
| rs2938134 | 17 | 50243397 | Intergenic | A | C | 0.327 | -0.018 | 0.003 | 3.14E-10 | -0.030 | 0.021 | 0.09 |
| rs2587507 | 17 | 77790135 | Intergenic | C | T | 0.498 | -0.015 | 0.003 | 8.69E-09 | -0.010 | 0.020 | 0.71 |
| rs34342129 | 18 | 5872472 | Intergenic | C | T | 0.491 | -0.014 | 0.003 | 2.13E-08 | 0.010 | 0.018 | 0.47 |
| rs4476253 | 18 | 25253297 | Intergenic | A | G | 0.24 | -0.018 | 0.003 | 5.78E-10 | 0.020 | 0.022 | 0.37 |
| rs7505855 | 18 | 31696075 | Intron: NOL4 | T | C | 0.414 | -0.017 | 0.003 | 5.31E-11 | 0.000 | 0.020 | 0.87 |
| rs8096225 | 18 | 36921851 | Intron: MIR924HG | C | A | 0.297 | 0.016 | 0.003 | 2.63E-08 | 0.030 | 0.021 | 0.12 |
| rs67050670 | 18 | 39297254 | Intergenic | G | A | 0.229 | -0.020 | 0.003 | 2.34E-11 | -0.010 | 0.023 | 0.60 |
| rs2359180 | 18 | 41314171 | Intergenic | G | A | 0.369 | -0.014 | 0.003 | 4.98E-08 | 0.000 | 0.020 | 0.92 |
| rs72898831 | 18 | 42658643 | Intergenic | G | A | 0.155 | -0.024 | 0.004 | 4.14E-12 | -0.010 | 0.025 | 0.83 |
| rs8083764 | 18 | 49874515 | Intron: DCC | T | G | 0.306 | -0.016 | 0.003 | 7.97E-09 | 0.020 | 0.020 | 0.34 |
| rs1373178 | 18 | 49967811 | Intron: DCC | G | T | 0.412 | -0.020 | 0.003 | 4.16E-15 | -0.010 | 0.020 | 0.69 |
| rs62098013 | 18 | 50863861 | Intron: DCC | A | G | 0.365 | 0.018 | 0.003 | 2.24E-11 | 0.010 | 0.023 | 0.54 |
| rs72938304 | 18 | 53661743 | Intergenic | A | G | 0.113 | -0.027 | 0.004 | 1.36E-11 | 0.030 | 0.032 | 0.30 |
| rs11872397 | 18 | 72535282 | Intron: ZNF407 | A | G | 0.253 | -0.017 | 0.003 | 5.20E-09 | 0.000 | 0.023 | 0.87 |
| rs71367544 | 18 | 77574374 | Intergenic | T | C | 0.203 | 0.021 | 0.003 | 8.54E-11 | -0.030 | 0.024 | 0.27 |
| rs10853981 | 19 | 4965064 | Intergenic | A | G | 0.33 | 0.015 | 0.003 | 4.88E-08 | 0.010 | 0.015 | 0.65 |
| rs113230003 | 19 | 18460956 | Intron: PGPEP1 | A | G | 0.255 | -0.019 | 0.003 | 1.05E-10 | -0.030 | 0.019 | 0.07 |
| rs8103660 | 19 | 18566395 | Intron: ELL | C | T | 0.354 | 0.016 | 0.003 | 3.03E-09 | -0.020 | 0.015 | 0.25 |
| rs117734003 | 19 | 51129745 | Intron: SYT3 | C | G | 0.0673 | 0.030 | 0.005 | 2.57E-09 | 0.000 | 0.023 | 0.87 |
| rs1126757 | 19 | 55879872 | Synonymous: IL11 | T | C | 0.473 | 0.014 | 0.003 | 2.92E-08 | -0.010 | 0.018 | 0.43 |
| rs6050446 | 20 | 25195509 | Nonsynonymous: ENTPD6 | G | A | 0.029 | 0.054 | 0.008 | 8.80E-13 | 0.010 | 0.062 | 0.88 |
| rs6058782 | 20 | 29946968 | Intergenic | T | C | 0.092 | 0.030 | 0.004 | 1.78E-11 | -0.010 | 0.028 | 0.72 |
| rs1555445 | 20 | 31175258 | Upstream: NOL4L-DT | T | A | 0.318 | 0.019 | 0.003 | 7.75E-12 | -0.030 | 0.022 | 0.12 |
| rs6073075 | 20 | 42015801 | Intergenic | A | T | 0.176 | -0.019 | 0.003 | 2.44E-08 | -0.020 | 0.029 | 0.52 |
| rs910912 | 20 | 54462393 | Intergenic | C | T | 0.261 | -0.017 | 0.003 | 7.82E-09 | 0.000 | 0.023 | 0.88 |
| rs6011779 | 20 | 61984317 | Intron: CHRNA4 | T | C | 0.194 | -0.019 | 0.003 | 2.83E-09 | -0.020 | 0.034 | 0.63 |
| rs3810496 | 20 | 62406886 | Intron: ZBTB46 | C | T | 0.381 | 0.016 | 0.003 | 1.54E-09 | 0.030 | 0.016 | 0.05 |
| rs4818005 | 21 | 40588819 | Intron: BRWD1 | A | G | 0.419 | -0.020 | 0.003 | 1.09E-14 | -0.030 | 0.018 | 0.18 |
| rs139896 | 22 | 38397797 | Intron: POLR2F | C | T | 0.352 | 0.015 | 0.003 | 7.14E-09 | 0.020 | 0.021 | 0.26 |
| rs4822102 | 22 | 42698430 | Intergenic | T | C | 0.382 | -0.017 | 0.003 | 2.78E-10 | -0.010 | 0.021 | 0.70 |
| rs9627272 | 22 | 46442288 | Intergenic | C | G | 0.407 | -0.015 | 0.003 | 2.42E-09 | -0.020 | 0.020 | 0.41 |

Abbreviations: Chr, Chromosome; RA, rheumatoid arthritis; SE, standard error; SNP, single nucleotide polymorphism.

| **Supplementary Table 2** The potential secondary phenotypes of the instrumental variables used for smoking initiation (from the GWAS catalog)^a^. | | | |
| --- | --- | --- | --- |
| SNP | Chr: Position | Trait^a^ | *P*-value |
|  |  |  |  |
| rs301807 | 1:8424763 | Sum eosinophil basophil counts | 3.00E-11 |
|  |  | Vitiligo | 4.00E-15 |
|  |  | Depression | 1.00E-10 |
| rs10914684 | 1:33329971 | Risk-taking tendency (4-domain principal component model) | 5.00E-09 |
| rs12740789 | 1:72286390 | Heart rate response to recovery post exercise (20 sec) | 1.00E-09 |
| rs10789369 | 1:73359226 | Schizophrenia | 4.00E-10 |
| rs1008078 | 1:90724174 | Educational attainment | 6.00E-41 |
| rs45444697 | 1:155062156 | Blood protein levels | 8.00E-15 |
| rs2901785 | 1:174135605 | Educational attainment (years of education) | 9.00E-10 |
| rs876793 | 1:237688783 | Lipid traits | 3.00E-07 |
| rs6731872 | 2:624205 | Alcohol consumption (drinks per week) (MTAG) | 4.00E-09 |
|  |  | Body fat distribution (arm fat ratio) | 2.00E-43 |
| rs2710634 | 2:32583737 | Educational attainment (MTAG) | 4.00E-08 |
|  |  | Highest math class taken (MTAG) | 2.00E-10 |
| rs1004787 | 2:44931952 | Alcohol consumption | 5.00E-32 |
| rs6730325 | 2:59088693 | Pulse pressure | 7.00E-10 |
| rs7585579 | 2:59797722 | Risk-taking behaviour | 3.00E-08 |
| rs11692435 | 2:97658891 | Alcohol consumption (drinks per week) (MTAG) | 3.00E-15 |
|  |  | Colorectal cancer | 1.00E-08 |
| rs11678980 | 2:161244750 | Self-reported math ability | 2.00E-23 |
|  |  | Highest math class taken | 4.00E-36 |
|  |  | Cognitive performance | 6.00E-25 |
| rs17229285 | 2:198658398 | Ulcerative colitis | 3.00E-14 |
| rs4674916 | 2:224500918 | Mathematical ability | 1.00E-08 |
| rs748832 | 3:16809700 | Intelligence (MTAG) | 2.00E-10 |
|  |  | Cognitive ability | 6.00E-11 |
| rs3172494 | 3:48694054 | Educational attainment (years of education) | 9.00E-11 |
| rs2276825 | 3:52852589 | Wellbeing measurement | 1.00E-12 |
| rs2306866 | 3:53732185 | Educational attainment (MTAG) | 2.00E-11 |
|  |  | Mathematical ability | 1.00E-11 |
| rs11128203 | 3:71015280 | Automobile speeding propensity (risk-taking behavior) | 1.00E-13 |
| rs62246017 | 3:71433933 | Squamous cell carcinoma | 1.00E-08 |
| rs74664784 | 3:85426142 | Alcohol consumption (drinks per week) | 2.00E-14 |
| rs2279829 | 3:147388532 | Alcohol consumption (drinks per week) (MTAG) | 1.00E-08 |
|  |  | Risk-taking behavior | 6.00E-09 |
|  |  | Number of sexual partners | 1.00E-13 |
| rs58400863 | 4:31182862 | Risk-taking behavior | 1.00E-15 |
| rs12517438 | 5:30841947 | Wellbeing measurement | 4.00E-08 |
| rs35375873 | 5:43190545 | Age at menarche | 4.00E-13 |
| rs72780746 | 5:104593887 | Number of sexual partners | 4.00E-10 |
| rs329124 | 5:134529762 | Self-reported educational attainment | 2.00E-09 |
|  |  | Body mass index | 2.00E-15 |
| rs1059490 | 6:26171022 | Mathematical ability | 9.00E-15 |
| rs3800227 | 6:108672958 | Anxiety measurement | 2.00E-08 |
| rs1737329 | 6:163386716 | Mathematical ability | 2.00E-10 |
| rs11780471 | 8:27487202 | Lung cancer | 2.00E-08 |
| rs11783093 | 8:27567832 | Schizophrenia | 8.00E-12 |
| rs6474609 | 9:10981069 | risk-taking behavior | 1.00E-09 |
| rs4877285 | 9:78739213 | Self-reported educational attainment | 3.00E-08 |
| rs2378662 | 9:84092374 | Age at menarche | 1.00E-11 |
| rs7026534 | 9:132031876 | Insomnia | 2.00E-08 |
| rs10858334 | 9:135097939 | Mathematical ability | 2.00E-10 |
| rs10905461 | 10:8761588 | Risk-taking behavior | 2.00E-09 |
| rs7072776 | 10:21744013 | Breast cancer | 1.00E-14 |
| rs28408682 | 10:102643553 | Waist-hip ratio | 3.00E-09 |
| rs12244388 | 10:102880295 | Risk-taking behavior | 1.00E-15 |
|  |  | Schizophrenia | 2.00E-18 |
| rs6265 | 11:27658369 | Body mass index | 7.00E-89 |
|  |  | Hip circumference | 1.00E-14 |
|  |  | Risk-taking behavior | 3.00E-10 |
|  |  | Visceral adipose tissue measurement | 2.00E-13 |
| rs4275621 | 11:28631449 | Attention deficit hyperactivity disorder | 3.00E-09 |
| rs1713676 | 11:113789854 | Alcohol consumption (drinks per week) | 4.00E-08 |
| rs1381287 | 14:98131215 | Risk-taking behavior | 1.00E-16 |
| rs1435741 | 15:47643646 | Risk-taking behavior | 3.00E-09 |
| rs11076962 | 16:5811367 | Mathematical ability | 7.00E-09 |
|  |  | Cognitive performance (MTAG) | 5.00E-11 |
|  |  | Self-reported educational attainment | 9.00E-10 |
|  |  | Intelligence | 3.00E-08 |
| rs7188873 | 16:24715743 | Intelligence | 2.00E-09 |
|  |  | Self-reported educational attainment | 5.00E-14 |
| rs4785187 | 16:49732861 | Self-reported educational attainment | 4.00E-10 |
| rs12918191 | 16:50911245 | Intelligence | 4.00E-08 |
| rs1050847 | 16:86943734-87943734 | Cognitive performance (MTAG) | 1.00E-10 |
|  |  | Self-reported educational attainment | 1.00E-12 |
| rs28441558 | 17:7899800 | Prostate cancer | 1.00E-16 |
| rs17692129 | 17:46715917 | Neuroticism | 5.00E-08 |
| rs62098013 | 18:53337491 | Multisite chronic pain | 4.00E-11 |
| rs10853981 | 19:4965053 | Triglycerides | 1.00E-11 |
| rs113230003 | 19:18350146 | Grip strength measurement | 2.00E-11 |
|  |  | Cardiovascular disease | 2.00E-08 |
| rs6011779 | 20:63352965 | Smoking behavior (cigarettes smoked per day) | 7.00E-10 |
| Abbreviations: Chr, chromosome; EA, effect allele; SNP, single nucleotide polymorphism. | | | |
| ^a^ Traits associated with the SNP according to previous genome-wide association studies. | | | |
